# Umbilical cord clamping and management of the third stage of labor: A telephone-survey describing Swedish midwives’ clinical practice

**DOI:** 10.18332/ejm/145697

**Published:** 2022-02-10

**Authors:** Manuela Isacson, Li Thies-Lagergren, Paola Oras, Lena Hellström-Westas, Ola Andersson

**Affiliations:** 1 Neonatology research group, Section of Pediatrics, Department of Clinical Sciences Lund, Faculty of Medicine, Lund University, Lund, Sweden; 2 Sachs' Children and Youth Hospital, Södersjukhuset, Stockholm, Sweden; 3 Midwifery research, reproductive, perinatal and sexual health, Department of Health Sciences, Faculty of Medicine, Lund University, Lund, Sweden; 4 Department of Obstetrics and Gynaecology, Helsingborg Hospital, Helsingborg, Sweden; 5 Department of Women’s and Children’s Health, Uppsala University, Uppsala, Sweden

**Keywords:** third stage of labor management, midwifery practice, prophylactic oxytocin, umbilical cord clamping, umbilical cord traction, vaginal delivery

## Abstract

**INTRODUCTION:**

The timing of cord clamping impacts children’s short- and long-term well-being. When making clinical decisions, midwives incorporate their tacit and professional knowledge, experience and current evidence. There appears to be a lack of knowledge regarding Swedish midwives’ management of the third stage of labor and cord clamping practice. The aim of this study was to explore Swedish midwives’ clinical practice concerning umbilical cord clamping and the third stage of labor in spontaneous vaginal births.

**METHODS:**

The study was designed as a cross-sectional telephone survey including 13 questions. Midwives were randomly selected from 48 births units in Sweden. Two midwives from each unit were interviewed. The primary outcome was timing of umbilical cord clamping practice in full-term infants. Secondary outcomes were the management of the third stage of labor including prophylactic use of synthetic oxytocin, the timing of cord clamping in preterm infants, controlled cord traction, uterine massage, and cord milking.

**RESULTS:**

Altogether, 95 midwives were interviewed. In full-term infants, all midwives preferred late cord clamping. Considerable heterogeneity was seen regarding the practices of synthetic oxytocin administration postpartum, controlled cord traction, uterine massage or cord milking, and cord clamping in preterm infants.

**CONCLUSIONS:**

Midwives in Sweden modify recommendations regarding delayed cord clamping in a way they might perceive as more natural and practical in their daily, clinical work. The study revealed a reluctance toward the administration of prophylactic oxytocin due to fear that the drug could pass to the infant. An overall large variation of the management of the third stage of labor was seen.

## INTRODUCTION

Since the 1940s, early cord clamping (CC), defined as clamping of the umbilical cord within 30 seconds after birth, has been the predominant practice globally^1^. Early CC became part of the Active Management of the Third Stage of Labor (AMTSL), as it is claimed to reduce the risk of postpartum haemorrhage^2,3^. However, there is increasing evidence that there may be short-term and long-term benefits for infants of delayed CC^4,5^. Increased early hemoglobin concentrations and iron stores in infants have been reported as well as improved neurodevelopment at two years of age compared to early CC. Possible disadvantages are polycythemia, jaundice, and increased requirement of phototherapy^4,5^.

Delayed CC is usually defined as clamping at least beyond the first minute after the birth of the infant even though the definition of ‘delayed’ in the literature has varied from 30 s to 5 min or to the cessation of pulsations in the umbilical cord^4^. The International Liaison Committee on Resuscitation^6^ and the American Academy of Pediatrics^7^ recommend delayed CC for longer than 30 s for both term and preterm infants. In term infants, the World Health Organization recommends delayed CC longer than 1 min^3^. The Swedish recommendations from 2008 state that CC should be delayed 2–3 min and this still prevails in Sweden^8^.

When making clinical decisions, Swedish midwives, in common with midwives in many other countries, incorporate their knowledge, experience and current evidence^9^. Midwives’ management of the third stage of labor seems to vary significantly throughout the world. An international pilot observation study^10^ and a European survey of policies found large differences in practices during the third stage of labor, both between and within countries^2^. According to a recent Cochrane report the practice of AMTSL, which involves prophylactic uterotonic, early CC and controlled cord traction (CCT) to reduce postpartum hemorrhage (PPH) in the third stage of labor, may need to be revised. Anyone taking on this task might also find it advisable to examine the desirability of more uniform practice within a country^11^.

In Sweden, midwives are the primary care providers during normal labor and birth. Their approach regarding CC is based on international, national and hospital guidelines, previous experience, and their own individual risk assessments^12^. A search in current literature revealed a lack of knowledge regarding Swedish midwives’ management of the third stage of labor and in particular regarding umbilical CC practice. This study aims to explore Swedish midwives’ clinical practice concerning umbilical cord clamping and the third stage of labor in spontaneous vaginal births.

## METHODS

A quantitative descriptive design was employed in this survey investigating midwives’ clinical practice postpartum with respect to birthing the placenta, umbilical CC and the use of synthetic oxytocin for control of blood loss.

### Setting and participants

Midwives working in the 49 birth units that existed in Sweden at the time the study was conducted, were contacted by telephone and invited to participate in a telephone survey. One unit, not existing today, had only a telephone number for emergency use and was excluded. The principal investigator (MI) called the birth unit and the midwife answering the phone was then asked if she was willing and able to participate. Inclusion was random but carried out according to predefined inclusion criteria. Midwives currently providing care during labor and birth were eligible and included if they agreed to participate. It was planned that two midwives in every birth unit should be included, that is, the units were contacted twice. When the second call was made, the investigator asked for confirmation that the midwife answering was not already part of the study.

### Study design and data collection

A cross-sectional telephone survey was performed between 1 October and 11 December 2015. To investigate midwives’ management of the third stage of labor and clinical practices regarding umbilical CC when caring for full-term as well as preterm infants, a semi-structured questionnaire including 13 questions designed with suggested categories of replies was constructed^13^. When the midwife more thoroughly explained the answer or the reason why a specific practice was performed the interviewer noted this in the margin of the questionnaire^13^. If needed, the question was clarified and sometimes the interviewer asked the midwife for clarification of the answer.

The questionnaire included questions about the following topics: 1) Current and previous routines and personal preferences, including policies/guidelines/routines and timing of CC in full- and preterm infants; 2) Policies/guidelines/routines and personal preferences regarding routines for timing, route and dose of synthetic oxytocin postpartum in full-term and preterm infants; and 3) Other practices, i.e. CCT, uterine massage, and cord milking. Data collection was performed through telephone interviews. Prior to conducting the interviews, the questionnaire was tested for face validity by two midwives to ensure that the questions were comprehensible. No modifications of the questions were needed, and the two pilot interviews were included in the results. The interviews lasted between 10–40 min.

### Statistical analyses

The data were analyzed and presented as descriptive statistics. Timing of CC in full-term and preterm infants, the use of CCT, uterine massage and cord milking during the third stage of labor, as well as dose, route and timing of administration of synthetic oxytocin, were reported. Some of the midwives’ more clarifying answers were used as citations in order to emphasize the quantitative data. Data were entered in Microsoft Excel for Mac (2011) and then imported into and analyzed with the Statistical Package for Social Sciences for Windows, version 22.0 (SPSS, Inc., Chicago, IL, USA).

### Ethical considerations

This study is based on the research done for an unpublished Master’s thesis. The research was assessed as not revealing sensitive personal data and therefore according to the Swedish Research Council (2020) did not need ethical approval. Before the start of every survey interview, participants were informed about the aim of the study, implementation and their rights as research participants. They then gave oral consent to participate in the survey. All the midwives who were approached could choose whether to answer or decline to answer a question, and could at any time during the interview stop the telephone survey. This was in accordance with the ethical principles for medical research involving human subjects as stated in Helsinki Declaration from World Medical Association (WMA, 2018).

## RESULTS

Data from 48 units represented by answers from 95 midwives were analyzed. All midwives were female. Of the 95 participating midwives, 76 worked in local/emergency hospitals and 19 in university hospitals. At one university hospital, only one midwife could be interviewed. Two other midwives declined to participate.

### Management of the third stage of labor

Overall, the strategies for managing the third stage of labor varied between midwives regarding one or more of the procedures mentioned earlier. Each midwife explained her approach to active management or expectant management. The following citations illustrate that practice varies from individual to individual. One respondent said:

*‘I know many are more active, but I think it is important to let the body do it, the best is if it happens in a natural way.’*

Another said:

*‘Actually, according to our guidelines, we should manage the third stage [of labor] actively but to be honest I do not.’*

Other midwives stated they had recently decided to use active management of third stage of labor and were currently in training to learn it, as illustrated by this reply:

*‘Well, I am very active – pull the cord and palpate the uterus all the time and I suppose that could be called massage.’*

### Clamping of the umbilical cord

Among the midwives who answered the survey, CC timing practices varied as illustrated in Table 1. All the midwives stated that they presently practiced delayed CC in uncomplicated, full-term, vaginal births. The majority of the midwives (n=57; 60%), stated that in full-term infants they clamped the cord after cessation of pulsations and some mentioned that the clamping time was probably more than 10 min in full-term births. Forty-seven midwives (49%) stated that they had previously clamped early; out of whom 41 had clamped early following established local or regional policies. These midwives changed to delayed CC when the policies were changed in 2008. The midwives in this study believed in the evidence recommending delayed CC, but they also noted that:

*‘Today most couples are aware of the benefits from delayed cord clamping, and ask for it, so that's another reason I delay the clamping.’*

**Table 1:** Swedish midwives’ replies regarding the preferred timing of umbilical cord clamping, in fullterm and preterm deliveries (N=95)

*Full-term infant (≥ 37+0 gestational weeks)*	*n (%)*
Within 30 s	0
30 s – 1 min	0
1–2 min	9 (9.5)
3–5 min	25 (26.3)
After cessation of pulsations	57 (60.0)
After the birth of the placenta	4 (4.2)
**Preterm infant** (<37+0 gestational weeks)	
Within 30 s	11 (11.6)
30 s – 1 min	13 (13.7)
1–2 min	9 (9.5)
3–5 min	20 (21.1)
After cessation of pulsations	34 (35.8)
After the birth of the placenta	0
Do not deliver preterm infants	8 (8.4)

Regarding preterm infants, the preferred clamping time was not as consistent as for full-term infants. The midwives pointed out that it was most important not to delay resuscitation and if the preterm infant was healthy, midwives would manage the third stage of labor as they did with full-term infants, although clamping time usually ended up being earlier.

### Synthetic oxytocin administration postpartum

Synthetic oxytocin was generally the only uterotonic drug used and the preferred dose, route and timing differed among midwives. Table 2 shows the timing, administration and dose of synthetic oxytocin used among the included 95 midwives. An example from one of the midwives not administrating synthetic oxytocin routinely was:

*‘I don't want to give it. It is not natural; it affects the infant negatively and it doesn't decrease severe maternal hemorrhage – so why should I give it?’*

**Table 2:** Swedish midwives’ replies on preferred timing, administration and dose of uterotonic postpartum (N=95)

*Timing of administration of uterotonic*	*n (%)*
Before umbilical cord clamping	67 (70.5)
After umbilical cord clamping	21 (22.1)
Usually do not give it	7 (7.4)
Preferred route of uterotonic administration*	
Intramuscular	10 (10.5)
Intravenous	63 (65.3)
No preferred route	22 (24.2)
Dose of synthetic oxytocin (uterotonic)*	
5 international units (IU)	39 (41.1)
10 international units (IU)	55 (57.9)

* One missing.

Among the 21 midwives who preferred administrating synthetic oxytocin after cord clamping, eight stated that they did this to avoid the risk that synthetic oxytocin would pass to the infant.

### Other practices postpartum

Controlled cord traction (CCT) was performed by 17 (18%) of the midwives. Midwives who replied they performed a gentler cord traction and then pulled only sometimes were coded as ‘sometimes’ performing CCT. None of the midwives practiced cord milking regularly but 19 (20%) performed it sometimes in preterm births, if the cord needed to be clamped early or after some minutes if it was a thick cord. Some midwives had never heard about cord milking.

### Policies, guidelines and routines

More than half of the midwives stated delayed CC was practiced according to a mostly unwritten understanding, but they formally agreed upon tradition in their units. Almost all reported that their birth unit had specific written guidelines regarding administration of synthetic oxytocin postpartum and most of the midwives adhered to the local guidelines. Some midwives pointed out, again, that they thought guidelines were unnecessary:

*‘Yes, we do have policies and guidelines but if you can motivate why you don't give it [synthetic oxytocin] – then it is Ok to not give it [synthetic oxytocin], and I rarely consider it [synthetic oxytocin] necessary.’*

Fourteen midwives stated that their hospital had overall guidelines regarding the management of the third stage of labor during normal, uncomplicated birth. In this study, the timing of cord clamping did not differ between timing reported by midwives adhering to guidelines and midwives not adhering to guidelines. It was unclear if all included labor wards had written policies regarding the timing of CC in preterm infants, but 24 (38%) reported available guidelines and clamped after one minute. Three midwives reported having no routine or did not know if there was a routine but did not clamp later than one minute.

## DISCUSSION

The main finding from this national telephone survey was that midwives most often reported waiting until pulsations had ceased and then clamped the umbilical cord. They were practicing delayed CC, which is consistent with results from contemporary studies suggesting there has been an increase in the practice of delayed CC over the last 20 years. Nevertheless, the studies report a significant variation in the waiting time before clamping the cord^14,15^. Possibly the transition toward delayed CC practice in Sweden was facilitated by the national guidelines that recommend delayed CC^8^. However, in our study, midwives reported a practice in part opposing the recommendations from 2008 stating that a delay of more than 3–5 min should be avoided^8^. Most often the midwives perform CC after pulsations have ceased. Delayed CC often takes place once cord pulsations have ceased^4^. Until now there is no evidence to recommend an upper limit regarding the timing of CC. However, even if the optimal timing of CC is still urgently required, it is claimed that early CC should be reframed as an intervention^16^.

Boere et al.^17^ report, based on an in vitro study, that the use of cessation of the pulsations as a time point for CC should be reconsidered. They observed that arterial flow can continue after the venous flow has stopped and that the cessation of umbilical cord pulsations was not correlated with the actual arterial flow. The authors suggest further studies to determine the optimal timing of clamping the cord^17^. Cord clamping after pulsations have ceased, and not after a specific period of time (e.g. 2–3 min), might be a result of attempting to find a balance between treating birth as a normal process and as a biomedical event^12^.

During the last decade, research has demonstrated the benefits for the neonate of delaying CC^18-20^, and this finding has led to a change in the strategies for timing of umbilical CC and cutting, in Sweden and several other countries as well^21^. Several barriers for implementation exist, but a multidisciplinary approach is needed for practice to be adopted^22^. When Leslie et al.^23^ explored the factors leading to change when midwives followed evidence-based recommendations (e.g. from early CC to delayed CC), five themes emerged as drivers of change: trusting colleagues, believing the evidence, honoring the preference of mothers and families, knowing personal certainty and protecting the integrity of the mother and the baby. Midwives included in the present study made comments that could be coded into these themes.

We also found that in Sweden there is a substantial variation in the overall management of the third stage of labor. This finding is consistent with results from previous studies showing a global variation in the use of AMTSL^10^. Festin^10^ reported in an international survey that midwives’ possible reluctance toward AMTSL was due to the fact that the use of AMTSL implied an active approach and they were thus forced to intervene. This is in line with the Jangsten et al.^12^ qualitative study of experienced midwives’ practices postpartum stating that midwives perceive birth as normal, and their practice aims to maintain birth as a physiological rather than a pathological process. A Canadian cross-sectional survey^24^ found that midwives were aware of AMTSL guidelines but that most of them rejected the recommendations as they felt the guidelines did not take into account the women’s preferences. Also, in a qualitative study carried out in Ireland and New Zealand, the approach described as ‘watchful waiting’ and/or ‘alert vigilance’ was followed by the midwives^11^. It is natural for midwives to embrace and respect women’s right to decision-making during labor and birth. An American retrospective explorative study^25^ found that approximately one in three of the total of 1069 women in the study declined to follow AMTSL. The timing of CC and the practice of AMTSL are closely connected. According to a Cochrane systematic review^11^ delayed CC may benefit the baby by blood transfusion, seen as a gain in birth weight. The review also states that it is not yet clear whether the intervention can be restricted to administering a uterotonic in order to reduce severe bleeding while excluding the other parts of AMTSL.

The use of CCT among midwives in this study was low, which is in line with Begley et al.^26^ who also reported this practice to be low. The contribution of CCT to decreasing PPH has been found to be minimal if synthetic oxytocin is given as a prophylactic treatment^27,28^. This indicates that the use of synthetic oxytocin as a prophylactic is sufficient in settings where there is ready access to midwives or other skilled attendants.

Also, our study found that the midwives were reluctant to routinely use prophylactic oxytocin postpartum. The reluctance towards synthetic oxytocin postpartum both in our study and in other studies raises a question on the future role of synthetic oxytocin postpartum. Many midwives replied that they managed the third stage expectantly even though they routinely administered synthetic oxytocin. This discrepancy between the stated expectant management and the frequency of components being part of active management could be due to unclear definitions of expectant versus active management within existing policies and educational programs. A Cochrane review published in 2010 recommended that prophylactic oxytocin postpartum should be administered after cutting the cord because not enough is known about the influence of synthetic oxytocin on the volume of placental transfusion and about any possible side effects on the newborn infant^29^. However, a recent randomized controlled trial by Vain et al.^30^ claims that administration of synthetic oxytocin after the birth of the infant but before CC does not modify the positive effects of delayed CC in healthy infants born at term. Yet, it is still uncertain if there are any adverse side-effects of using prophylactic oxytocin with an intact umbilical cord.

In Sweden, the lack of a concurrent way of managing the third stage of labor, together with the high rate of synthetic oxytocin administration postpartum may indicate that midwives are following the national guidelines from 2001, where 10 IU oxytocin intravenous was recommended immediately after the birth of the infant^31^ but neither AMTSL nor any other management is recommended or dismissed. Currently, there are no national guidelines regarding the administration of synthetic oxytocin postpartum except that practice is to be decided by staff in each region or local hospital. Also, it makes it difficult to differentiate between synthetic oxytocin administered intrapartum or postpartum, which is most likely to give rise to different effects^32^. The non-adherence to AMTSL seems to be global and should be further evaluated.

### Strengths and limitations

A telephone survey was chosen as the method for data collection because the slow and low response rate of mail surveys was considered a limitation^33^. Also, a telephone interview provides a more personal contact and makes it possible for the interviewer and/or respondent to ask for clarification. The telephone survey was especially well suited for this study since the study population was dispersed over a large geographical area^33^.

There are several limitations to be acknowledged. The respondents at each birth unit do not represent all midwives at any one birth unit and responses differed between midwives working in the same birth unit, limiting the generalizability of the results. Also, background information such as the respondent’s age and the number of years working as a midwife is lacking. The study relied on self-reported practice and we cannot know if each respondent actually practiced as stated. Variation in clinical practice from the south to the north of the country was evident, illustrating that even within this single country there is significant variation.

It may be that the questions regarding guidelines probably were interpreted in different ways. Some midwives stated they had guidelines if the birth unit had a strong verbal routine while others, with the same preconditions, might have thought the question referred only to a specific printed document and therefore answered they did not have guidelines.

Despite these limitations, no uniformity in the management of the third stage of labor was found, a finding common in other studies^34-36^. Even though the study was not a mixed-methods study, some samples of citations were included in order to provide a useful supplement to the descriptive data.

## CONCLUSIONS

In a limited sample in Sweden, midwives practiced delayed CC when assisting births of full-term infants. The study indicates that the midwives have modified the recommendations of delayed CC in a way they might perceive as more natural and practical in their daily, clinical work, that is after cessation of pulsations. As an incidental finding, reluctance toward administration of prophylactic oxytocin was observed and a fear that the drug could pass to the infant was expressed. Also, a large variation in the overall management of the third stage of labor was seen. Our findings highlight a need to explore midwives’ decision process regarding the management of the third stage of labor and cord clamping, preferably at more depth by qualitative methods.

## Data Availability

The data supporting this research are available from the authors on reasonable request.
